# An asymmetry of translational biological motion perception in schizophrenia

**DOI:** 10.3389/fpsyg.2013.00436

**Published:** 2013-07-16

**Authors:** Caitlín N. M. Hastings, Philip J. Brittain, Dominic H. ffytche

**Affiliations:** ^1^Department of Old Age Psychiatry, Institute of Psychiatry, King’s College LondonLondon, UK; ^2^Department of Psychological Medicine and Psychiatry, MRC Social, Genetic and Developmental Psychiatry Centre, Institute of Psychiatry, King’s College LondonLondon, UK

**Keywords:** functional outcome, motion perception, STS, social cognition, translational motion, fMRI

## Abstract

**Background:** Biological motion perception is served by a network of regions in the occipital, posterior temporal, and parietal lobe, overlapping areas of reduced cortical volume in schizophrenia. The atrophy in these regions is assumed to account for deficits in biological motion perception described in schizophrenia but it is unknown whether the asymmetry of atrophy found in previous studies has a perceptual correlate. Here we look for possible differences in sensitivity to leftward and rightward translation of point-light biological motion in data collected for a previous study and explore its underlying neurobiology using functional imaging.

**Methods:** *n* = 64 patients with schizophrenia and *n* = 64 controls performed a task requiring the detection of leftward or rightward biological motion using a standard psychophysical staircase procedure. six control subjects took part in the functional imaging experiment.

**Results:** We found a deficit of leftward but not rightward biological motion (leftward biological motion % accuracy patients = 57.9% ± 14.3; controls = 63.6% ± 11.3 *p* = 0.01; rightward biological motion patients = 62.7% ± 12.4; controls = 64.1% ± 11.7; *p* > 0.05). The deficit reflected differences in distribution of leftward and rightward accuracy bias in the two populations. Directional bias correlated with functional outcome as measured by the Role Functioning Scale in the patient group when co-varying for negative symptoms (*r* = -0.272, *p* = 0.016). Cortical regions with preferential activation for leftward or rightward translation were identified in both hemispheres suggesting the psychophysical findings could not be accounted for by selective atrophy or functional change in one hemisphere alone.

**Conclusion:** The findings point to translational direction as a novel functional probe to help understand the underlying neural mechanisms of wider cognitive dysfunction in schizophrenia.

## INTRODUCTION

Visual function has long been recognized as altered in schizophrenia (Silverstein and Keane, 2011). Motion perception is one aspect of vision affected, with differences between patients and controls reported for motion coherence, velocity and luminance contrast (for review see Chen, 2011). There is an apparent hierarchy of motion effects within schizophrenia with global motion more affected than local motion (Chen et al., 2003) and biological motion more than global motion (Brittain et al., 2010a). Furthermore, at the top of the motion hierarchy, biological motion appears linked to an important outcome measure – the level of social functioning. Patients with poorer biological motion perception have less favorable social outcome, biological motion sensitivity correlating directly with social outcome (Kim et al., 2005) or indirectly through social perception (Brittain et al., 2010a). The neurophysiology, brain networks and psychophysics of motion perception are well understood, providing a useful model system from which to approach the underlying neurobiology of wider cognitive dysfunction schizophrenia (Chen, 2011; Silverstein and Keane, 2011). In its link to social functioning, biological motion is of particular interest in this regard.

Motion perception involves a network of regions in the occipital, posterior temporal, and parietal lobes. In the occipital lobe, the primary visual cortex and its immediate surrounds (areas V1 and V2) respond to all classes of motion (Watson et al., 1993) while different sub-regions of the lateral surface of the occipital and posterior temporal lobes respond to different classes of motion. Biological motion is a term used for a class of motion first characterized by Johansson (Johansson, 1973) in which walking or movements such as jumping, running, kicking, throwing, crawling, shoveling, dancing are defined by point-light sources. Such stimuli have attracted considerable research interest due to their inherent combination of motion, form and action that may help reveal how such properties are integrated in the brain. The key brain regions implicated in previous studies are: (i) the posterior superior temporal gyrus (STG) and cortex surrounding the superior temporal sulcus (STS), bilaterally in some studies and predominantly right hemispheric in others (Bonda et al., 1996; Howard et al., 1996; Vaina et al., 2001; Peuskens et al., 2005; Peelen et al., 2006) (ii) regions in the ventral temporal lobe overlapping or in close relation to regions involved in face, object, figure, and kinetic contour processing (Vaina et al., 2001; Grossman and Blake, 2002; Peuskens et al., 2005) (iii) the cerebellum (Vaina et al., 2001; Sokolov et al., 2012) (iv) frontal cortex (Vaina et al., 2001; de Lussanet et al., 2008) and (iv) the parietal lobe (Bonda et al., 1996; Vaina et al., 2001).

It is assumed that functional or structural changes in the networks described above underlie the psychophysical motion deficits found in schizophrenia, a view supported by the finding that differences in functional activation for hits, false alarms and correct rejections in the posterior STS for biological motion stimuli differ in patients with schizophrenia from controls (Kim et al., 2011). Brain lesions in the parietal lobe/parieto-temporal junction (Battelli et al., 2003), superior temporal or inferior frontal regions (Saygin, 2007) and anterior temporal lobe (Vaina and Gross, 2004) are associated with deficits in biological motion. These regions typically have reduced cortical volume in structural imaging studies of schizophrenia. Although imaging findings vary from study to study, Shenton et al. (2001) in a review of the literature found 15/15 studies reporting a decrease in STG gray matter volume. Similarly, 9/15 studies reported volume reductions in the parietal lobe and 30/50 studies in the frontal lobe, particularly prefrontal cortex.

A consistent finding in structural imaging studies of schizophrenia is an asymmetry of atrophy in the left and right hemispheres. Within the network of areas linked to biological motion, the left STG is typically more affected than the right (Shenton et al., 2001). Similarly, the left inferior parietal lobule is typically more affected than right inferior parietal lobule (Niznikiewicz et al., 2000). The question therefore arises as to whether the asymmetry in hemispheric atrophy has a perceptual correlate. One aspect of motion perception that seems to be represented differently in each hemisphere is the direction of translational motion – the movement of an object or dot pattern across the visual field. Unlike primary visual cortex, which responds to stimuli in the contralateral hemifield only, motion specialized cortex (area V5) responds to motion in both contralateral and ipsilateral fields through interactions between the two hemispheres (Tootell et al., 1998; ffytche et al., 2000). Motion specialized areas thus respond to movement across the whole visual field, with evidence to suggest a bias of representation in each hemisphere. Patients with left hemispheric lesions have a predominance of leftward motion perception deficits while patients with right hemispheric lesions have a predominance of rightward motion perception deficits (Barton et al., 1995). This suggests a relative specialization for leftward translational motion in the left hemisphere and rightward translational motion in the right hemisphere. Evidence from an intraoperative study disrupting motion specialized areas in the right hemisphere through stimulation resulted in predominantly rightward motion perception deficits (Blanke et al., 2002), consistent with this view.

To date, most studies of biological motion have used stimuli that remain fixed, without translation across the visual field (i.e., a figure walking in place as if on a treadmill). It is therefore unclear whether leftward translational biological motion is linked to the left hemisphere and rightward translational biological motion to the right hemisphere, as seems to be the case for coherent motion. However, the existence of an asymmetry is hinted at by studies of biological motion figures *facing* leftward or rightward while walking in place. Leftward-facing figures walking in place in the left hemifield are associated with greater activation of right hemispheric frontal and parietal regions than rightward-facing figures. Similarly, rightward-facing figures walking in place in the right hemifield are associated with greater activation in left hemispheric frontal and parietal regions than leftward-facing figures (de Lussanet et al., 2008). Leftward and rightward facing figures are also represented in spatially distinct sub-regions of the fusiform gyrus (Michels et al., 2009). Such findings lend support to the possibility of a difference in the representation of leftward and rightward translational biological motion in each hemisphere.

If leftward and rightward translation of biological motion are represented differently in each hemisphere, then asymmetrical atrophy within the biological motion network described in previous studies may be reflected as a difference in sensitivity to leftward and rightward biological motion translation. We have sought evidence to support this view using data from our previous study in which we found reduced sensitivity in schizophrenia to biological motion translation direction (Brittain et al., 2010a). Here we re-examine this data to establish whether the reduction in sensitivity identified related to one direction more than the other and report preliminary functional imaging evidence of the neurobiology of translation direction for biological motion stimuli.

## MATERIALS AND METHODS

Full details of patient recruitment and testing methods for the psychophysical study have been presented elsewhere (Brittain et al., 2010a, b). Patients with a DSM-IV diagnosis of schizophrenia (*n* = 64) were recruited from outpatient and inpatient facilities in South London and controls (*n* = 64) from local advertisement and a volunteer database. The study was approved by the Institute of Psychiatry Ethical committee and all subjects gave informed, written content. The two groups were matched for age, gender, level of education, visual acuity, and handedness but differed in IQ (estimated with the two-subtest version of the Wechsler Abbreviated Scale of Intelligence WASI, Psychological Corporation, 1999; patients = 101.91 ± [SD]15.24; controls = 107.37 ± 13.49). The inclusion criteria for both participant groups were: age between 18 and 65, English as a first language, no current alcohol or drug dependency, predominantly right handed (assessed using a six item version of the Annett Handedness Questionnaire Annett, 1970), no history of electroconvulsive therapy (none in the past 3 years for the patient group), no significant ophthalmological disease, sensory disability, history of epilepsy, or known neurological condition. Other tests performed of relevance to the analyses reported here are: (i) *Role Functioning Scale* (Goodman et al., 1993). This assesses functional outcome in four domains (working productivity, independent living/self-care, immediate social network relationships, extended social network relationships) with scores ranging from one (severely impaired) to seven (optimal). The Global Role Functioning Index (GFI) is the sum of the domain scores ranging from 4 (worst functioning) to 28 (best functioning) (ii) *Positive and Negative Syndrome Scale* (PANSS Kay et al., 1987) assessing positive, negative and general psychopathology symptoms in separate sub-scales.

### BIOLOGICAL MOTION TEST

An array of 50 randomly moving white dots appeared in a square area subtending 10° of visual angle on a black background. Each trial lasted 3500 ms. At a random time after trial onset, 12 of the dots moved as a biological motion array forming a figure walking at 4.5°/s either leftward or rightward. The figure could appear at any position in the screen at the onset of the trial. When the figure reached the left or right vertical edge of the square array of dots it re-appeared at the opposite edge. Subjects were not required to maintain fixation. At the end of the trial, participants were asked to respond whether the figure had moved (i) leftward, (ii) rightward or (iii) was not seen. Responses were logged by the experimenter. A correct response increased the number of randomly moving dots by 20 for the next trial (increment = 10 after first incorrect response). An incorrect or “not seen” response resulted in a decrease of 10 dots for the next trial. This resulted in a psychophysical staircase function that reached a plateau after approximately 15 trials in each block. Two blocks of forty-two trials were performed for each subject. Each block contained 21 leftward trials and 21 rightward trails in pseudorandom order (i.e., the number of trials for each direction was fixed but their order of presentation randomised) so that each trial (i.e., each point on the psychophysical staircase) had equal probability of being leftwards or rightwards. All subjects were trained on the task prior to testing and confirmed they were able to see the walking figure.

### ANALYSIS

The trials were sorted into leftward and rightward directions (42 trials for each direction in the two blocks combined) and an accuracy score for each direction derived for each subject. The leftward accuracy score = (number of correctly identified leftward trials / 42) × 100. The rightward accuracy score = (number of correctly identified rightward trials / 42) × 100. Trials with “did not see” responses were deemed incorrect. We also derived an accuracy score for the subset of trials at the plateau of the psychophysical staircase where the level of distractor dots was approximately constant. This threshold accuracy value related to the last 28 trials in each block and, because of the randomization of direction, varied from subject to subject in the total number of leftward and rightward trials. For each direction, group differences in accuracy score between patients and controls were tested using two-sample t tests. ANOVA models were used to examine the effects of gender and degree of right handedness. Within-subject measures of leftward and rightward accuracy were compared in a repeated measures ANOVA model with within-subject factor direction(left, right) and between-subject factor group(patient, control). Correlations between accuracy and functional outcome were explored using non-parametric tests (Spearman’s Rho, one-tailed tests) and parametric tests (Pearson’s) when co-varying for negative symptoms. Correlations between leftward and rightward accuracy and with IQ were measured using parametric tests (Pearson’s).

### FMRI METHODS

Six control subjects without history of neurological or psychiatric illness took part in the study (two male, four female; mean age 30 ± 6 years). All had normal corrected visual acuity and gave informed consent. Subjects were presented the same translational biological motion stimulus as used in the psychophysical study, with timings adapted for fMRI (8 s trial length with the stimulus appearing at a random time around 4 s after the appearance of distractor dot noise; inter-trial interval = 8 s) and a fixed number of distractors determined for each subject prior to the scan to standardize performance at ~70% correct. Subjects were not required to maintain fixation. Biological motion trials were interleaved with trials of coherent motion, optic flow and blank trials. Only data from biological motion trials is presented here (14 trials for each subject, seven leftward, seven rightward). Subjects responded with a right hand button press to indicate whether they had seen rightward, leftward or no motion.

### MRI ACQUISITION AND ANALYSIS

Functional images were acquired on a 1.5 Tesla GE Neuro-optimised Signa LX Horizon System (General Electric, Milwaukee, WI, USA), using a gradient echo planar sequence sensitive to blood oxygenation level dependent (BOLD) contrast (TR = 2 s; TE = 40 ms; flip angle 90°; 64 × 64 matrix; in-plane voxel size 3.75mm × 3.75 mm). 16 axial slices, 7 mm thick with 0.7 mm interslice gap, were acquired every 2 s. For each subject, the functional time series was motion corrected (Friston et al., 1996), transformed into stereotactic space and smoothed with a 7 mm FWHM Gaussian filter using SPM software (). The activity at each voxel was high-pass filtered and modeled by three covariates (distractor dot onset, leftward biological motion onset for correct trials; rightward biological motion onset for correct trials), convolved with the hemodynamic response function. Group activation maps for leftward > rightward and rightward > leftward translational biological motion were created using a fixed effect model that included all subjects.

## RESULTS

As described previously for the biological motion task, control subjects had a higher number of distractor dots at threshold than patients (number of distractor dots at threshold controls = 211.95 ± 63; patients = 186.25 ± 61; Brittain et al., 2010a). The level of distractor dots at threshold reflects the number of errors made in the staircase and one would expect, therefore, a significant correlation between accuracy and the number of distractor dots at threshold (*r* = 0.845, *p* < 0.001 for the group as a whole, the correlation is not perfect due to the varying position on the staircase of the first error and its associated change in step size). The issue we explore here is whether accuracy across the staircase differed for one direction and the other or, put another way, whether the reduced sensitivity in schizophrenia overall was driven primarily by reduced sensitivity in a single direction.

**FIGURE 1:**
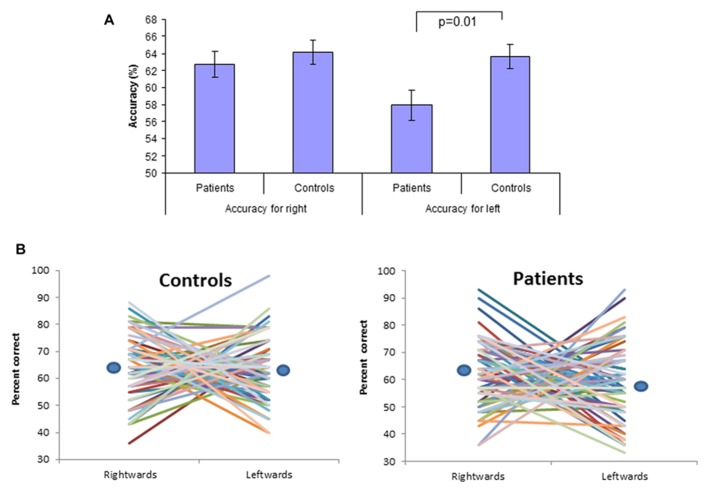
** Biological motion direction accuracy.** **(A)** accuracy scores for leftward and rightward motion for the cohort as a whole with a significant difference in accuracy for leftward motion only. **(B)** The accuracy scores for leftward and rightward directions in each subject are connected by a line to illustrate their negative correlation. The circles indicate the position of the mean values given in **Figure 1A**.

The accuracy results from each direction in all participants are illustrated in **Figure 1A**. There was no significant difference in accuracy between patients and controls for rightward motion (patients = 62.7% ± 12.4[SD]; controls = 64.1% ± 11.7; *t*_126_ = 0.66; *p* > 0.05). In contrast, a significant difference was found for leftward motion (patients = 57.9% ± 14.3; controls = 63.6% ± 11.3; *t*_126_ = 2.49; *p* = 0.01). The reduction in leftward motion accuracy was not influenced by gender (*F*_1,124_ = 0.75; *p* > 0.05) or right/ambiguous handedness (*F*_1,123_ = 1.16; *p* > 0.05). It does not reflect a bias in the patient group to respond “right” for trials that they were unsure of rather than using the “did not see” option as the average number of trials reported as “did not see” was similar in the two groups (patients = 28.9%, controls = 28.4%). These accuracy values relate to all trials in the staircase and thus contain a mixture of easy trials presented at the beginning of a block when the number of distractor dots is low and difficult trials presented at the end of a block when the number of distractor dots is high. We found the same pattern of results when examining the subset of difficult trials at threshold, although the level of significance was lower given the smaller number of trials and variability in the number of leftward and rightward trials used to derive the accuracy value (rightward motion at threshold patients = 56.2% ± 13.6; controls = 56.9% ± 14.1; *t*_126_ = 0.297; *p* = 0.76; leftward motion at threshold patients = 49.9% ± 16.7; controls = 55.1% ± 14.2; *t*_126_ = 1.86; *p* = 0.06). We also examined whether the accuracy values might be related to IQ. Treating the patients and controls as a single group, IQ correlated with leftward accuracy (*r* = 0.186; *p* = 0.01; higher IQ better accuracy score), but not rightward accuracy (*r* = -0.81; *p* = 0.18). The association with leftward accuracy was also found for the control group considered alone (*r* = 0.206; *p* = 0.05) and a negative correlation was found between rightward accuracy and IQ (higher IQ lower accuracy, *r* = -0.244; *p* = 0.02). In the patient group neither leftward accuracy (*r* = 0.113; *p* = 0.18) or rightward accuracy (*r* = 0.033; *p* = 0.39) correlated with IQ.

The reduction in leftward accuracy observed in the patient group is more complex than implied by the average data. There was a significant *negative* correlation between leftward and rightward accuracy for both patients and controls (controls *r* = -0.444, *p* < 0.001; patients *r* = -0.468, *p* < 0.001) such that, for most subjects, accuracy for one direction was better than the other. This relationship is illustrated in **Figure 1B** where a line is drawn for each subject connecting their leftward and rightward accuracy scores. While some subjects have approximately equivalent accuracy for leftward and rightward directions (horizontal lines), the majority have an asymmetrical bias (diagonal line). We derived an index of directional bias for each subject to further explore this issue (rightward accuracy – leftward accuracy; > 0 = rightward bias, < 0 = leftward bias; 0 = no bias). In controls, mean leftward bias was 17% ± 11 and mean rightward bias was 18% ± 11 with the magnitude of leftward and rightward bias balancing out such that, overall, mean accuracy was equivalent for leftward and rightward directions. In the schizophrenia group the distribution of bias was such that the rightward bias outweighs the leftward bias (mean leftward bias 18% ± 14; mean rightward bias 21% ± 12), with a consequent overall reduction in mean leftward accuracy. This effect is hidden in the within-subject ANOVA (group by direction interaction *F*_1,126_ = 1.285, *p* = 0.26) due to the high variance of accuracy difference across subjects with leftward and rightward direction bias. For the same reason, the within-subject *t*-test of leftward v rightward accuracy in the patient group is only at trend significance (patients mean difference 4.7% ± 23, *t*_63_ = 1.66, *p* = 0.10; controls mean difference 0.48% ± 20, *t*_63_ = 0.197, *p* = 0.84).

We next examined whether the directional bias index was linked to functional outcome. For the patient group as a whole there was a trend significant association for the functional outcome total score (rho = -0.175, *p* = 0.083) but significant and trend significant correlations with subscales of working productivity (rho = -0.208, *p* = 0.049), independent living/self-care (rho = -0.194, *p* = 0.063) and immediate social network relationships (rho = -0.173, *p* = 0.086). PANSS negative symptoms were strongly associated with functional outcome (rho = -0.577, *p* < 0.001) and controlling for negative symptoms, the relationship between directional bias index and functional outcome was strengthened (total score *r* = -0.272, *p* = 0.016; working productivity *r* = -0.225, *p* = 0.038; independent living/self-care *r* = -0.212, *p* = 0.047 and immediate social network relationships *r* = -0.235, *p* = 0.032).

### FMRI RESULTS

**FIGURE 2:**
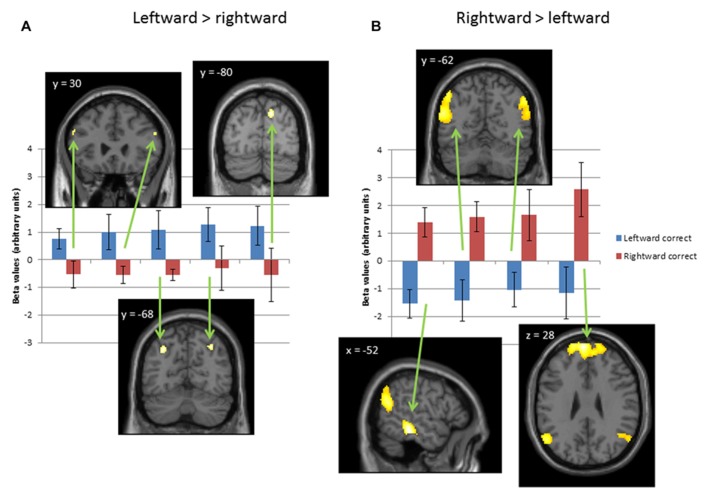
** Brain regions selectively activated by leftward or rightward translational biological motion.** Coronal, axial, and sagittal slices of the SPM single subject canonical image is shown (MNI x, y or z co-ordinate indicated for each slice) through regions of activation in the group analysis for: **(A)** leftward translation greater than rightward translation; **(B)** rightward translation greater than leftward translation. Threshold *p* < 0.05 uncorrected, 10 contiguous voxels. The graphs show mean and standard error beta values in regions indicated by the arrows for correctly identified leftward (blue) and rightward (red) motion trials.

Of the six subjects taking part in the fMRI study, four had leftward bias on the asymmetry index (32 ± 14%) and two had rightward bias (43 ± 0%). Pooling both sets of subjects we identified regions activated more for leftward than rightward translation of biological motion and vice versa. Given the exploratory nature of the study and small number of subjects and trials, a lenient threshold of *p* < 0.05 uncorrected and 10 contiguous voxels was used. **Figure 2** shows regions activated at this threshold by leftward motion (blue bars) more than rightward motion (red bars; **Figure 2A**) or rightward motion more than leftward motion (**Figure 2B**). Areas preferentially activated by leftward motion included bilateral regions of dorsolateral prefrontal cortex (MNI co-ordinates ± 56 30 28) bilateral regions in the intra-parietal sulcus (MNI co-ordinates ± 32 -68 40) and right cuneus (MNI co-ordinates 14 -80 40). Areas preferentially activated by rightward motion included bilateral regions in the supramarginal gyrus (MNI co-ordinates ± 52 -62 22), left STS/middle temporal gyrus (MNI co-ordinates -54 -32 -14) and bilateral medial frontal regions (MNI co-ordinates ± 4 60 28). The pattern of preferential leftward and rightward activation was the same when the analysis was restricted to the four subjects with leftward bias alone. The number of subjects with rightward bias was too small to draw any conclusions as to whether regions of preferential activation differed in this subgroup.

## DISCUSSION

We have sought evidence for an asymmetric sensitivity to direction of biological motion translation in patients with schizophrenia based on an asymmetry of atrophy within regions linked to biological motion found in previous studies. Although we found evidence in support of an asymmetry, the findings suggest a more complex relationship between direction and hemisphere than envisaged. Below we discuss the findings in the light of preliminary functional imaging evidence and explore their wider implications.

### LEFTWARD AND RIGHTWARD DIRECTION DISCRIMINATION

Although motion speed, coherence, local/global features and direction have been studied extensively in schizophrenia (see Chen, 2011 for review), as far as we are aware no studies have reported thresholds for leftward and rightward motion separately. Where direction discrimination has been investigated in previous studies (e.g., Chen et al., 2003; Slaghuis et al., 2005; Brittain et al., 2010a) the methods used measure overall performance on a leftward/rightward discrimination task rather than thresholds for leftward and rightward directions separately. The apparent deficit in leftward motion reported here is therefore an entirely novel finding. We were unable to explore whether it is also apparent in the global coherent motion task reported in our previous studies (Brittain et al., 2010a, b) as the coherent motion task involved upward and downward, not leftward and rightward directions.

The interpretation of the leftward direction deficit found in schizophrenia is more complex than anticipated. Unexpectedly, sensitivity for leftward and rightward directions were negatively correlated in the group as a whole, with the deficit in schizophrenia reflecting a difference in relationship between leftward and rightward accuracy rather than a deficit of sensitivity for leftward translation alone. As far as we are aware, a negative correlation of leftward and rightward direction sensitivity has not been reported before. It is important to note that our analysis is retrospective, based on previously collected data, and uses a non-conventional analysis of a standard psychophysical staircase. The analysis potentially introduces systematic biases as, for the ideal observer at the staircase plateau that defines threshold, successive trials may be seen and not seen in alternation because the number of distractor dots alternately increases and decreases. If the randomization of directions in the plateau allocates alternating leftwards and rightwards trials, one direction would be seen and the other not. However, this chance occurrence would not favour one direction over the other, i.e., could equally be “left seen, right not seen” as “right seen, left not seen”. Any small bias in one direction for a block of trials would even out across repeated blocks and through combining data from different subjects. Furthermore, longer sequences of alternating directions lead to higher % accuracy for one direction but have no effect on % accuracy for the other so that such biases do not account for the negative correlation between directions found. It therefore seems unlikely our results can be explained by the non-conventional nature of our analysis. Indeed, the use of a single staircase to compare accuracy for the two directions would be more likely to introduce a spurious positive correlation between directions than the negative correlation found. The negative correlation of direction accuracy is also not explained by systematic differences in reporting in the two groups. IQ in the patient group was lower than in the control group and thus one might argue the patient group had greater difficulty understanding the task. However, while this might account for an overall reduction in accuracy, it seems unlikely that it could account for a deficit in one direction of translation but not the other. Similarly, one might argue that the patient group had medication and psychopathology not present in the control group that could influence performance in the task but it seems improbable that such effects could impact on one direction only. Sensitivity to biological motion is influenced by a number of factors including size and eccentricity (Gurnsey et al., 2008), body part (Takahashi et al., 2011), facing direction for stimuli presented in a given hemifield (de Lussanet et al., 2008) and executive control (Chandrasekran et al., 2010). However, apart from facing direction, these factors were identical for leftward and rightward translation so it is difficult to account for differences in sensitivity for the two directions in terms of these factors. The leftward translating stimulus was presented as if facing left and the rightward translating stimulus presented as if facing right; however, the trajectory of the walking figure crossed both left and right hemifields so that differential sensitivity to facing direction in one hemifield would be offset by the opposite sensitivity in the other hemifield. In support of this view, we did not find differential activation in our study within sub-regions of the fusiform gyrus sensitive to facing direction (Michels et al., 2009). In summary, although the underlying mechanism of directional bias and leftward accuracy deficit in schizophrenia requires further investigation, it does not seem to be accounted for by non-specific differences between the patient group and controls or by known factors influencing sensitivity to biological motion.

Our fMRI analysis was exploratory and used a lenient threshold in which many of the regions identified would not survive correction for multiple comparisons. However, it provides clues as to the types of functional or structural change in schizophrenia that could underlie the psychophysical findings. Importantly the fMRI findings suggest that our hypothesis of preferential representation for leftward motion in the left hemisphere and rightward motion in the right hemisphere derived from the coherent motion literature is over simplistic for biological motion. The areas identified in this study are predominantly bilateral so it is unlikely that any differences in structure or function in patients with schizophrenia restricted to one hemisphere would cause the shift in bias and consequent decrease in leftward accuracy found in the psychophysical data. What is more likely is that, in schizophrenia, functional changes in bilateral subsets of regions, for example decreased activity in bilateral dorso-lateral pre-frontal cortex or increased activity in bilateral STG, is responsible for the psychophysical changes. The fMRI data also raises the intriguing possibility that the negative correlation of leftward and rightward accuracy described in the psychophysical data might be linked to the reciprocal relationship of leftward and rightward responses within brain areas.

### DIRECTIONAL BIAS AND FUNCTIONAL OUTCOME

Why might functional outcome be linked to a bias in translational direction perception? It seems unlikely the small overall deficit in leftward direction (a decrease in accuracy of 5% in the patient group) would have specific effects on social function. What seems more probable is that directional bias is an indirect measure of wider cognitive functions including (i) theory of mind cognition, linked in previous studies to motion perception (Kelemen et al., 2005) or (ii) the comprehension of action movements (Bonda et al., 1996). The unexpected association of IQ with leftward accuracy in the cohort as a whole and with leftward and (negative) rightward accuracy in the control group lends support to this view. We assume an aspect of social cognition or wider cognitive function, co-localized or localized in close proximity to regions underlying directional bias, are responsible for these associations. The correlation coefficient linking functional outcome to directional bias is higher than that between functional outcome and biological motion threshold although the difference is not statistically significant (*r* = 0.129 for threshold versus outcome, *r* = (-)0.272 for directional bias versus outcome, co-varying for negative symptoms in both tests, *p* = 0.3 in Z transform test). In contrast, both these associations with functional outcome are lower than that reported by Kim et al. (2005) for biological motion sensitivity (*r* = 0.7; difference *p* = < 0.01 Z transform test). However, the Kim et al. (2005) study used a measure of outcome weighted by age and education which might account for the higher correlation.

## CONCLUSION

The deficit in biological motion perception for leftward translation we have identified in patients with schizophrenia and its link to functional outcome remains unexplained. However, it points to direction of translation sensitivity as a potentially important area of investigation in schizophrenia. The results presented here suggest measures of leftward and rightward biological motion translation may help explore cortical function in key frontal, parietal and temporal regions serving social cognitive function and their interaction across hemispheres to better understand the neurobiology of cognitive change in schizophrenia.

## Conflict of Interest Statement

The authors declare that the research was conducted in the absence of any commercial or financial relationships that could be construed as a potential conflict of interest.
